# Promoting proliferation and differentiation of BMSCs by green tea polyphenols functionalized porous calcium phosphate

**DOI:** 10.1093/rb/rbx031

**Published:** 2017-12-11

**Authors:** Kang Zhou, Xiuli Ren, Mengen Zhao, Xifan Mei, Peng Zhang, Zhenhua Chen, Xiangdong Zhu

**Affiliations:** 1 Jinzhou Medical University, Jinzhou 121001, People’s Republic of China and; 2 National Engineering Research Center for Biomaterials, Sichuan University, Chengdu 610064, People’s Republic of China

**Keywords:** green tea polyphenols, porous ceramics, BMSCs, osteoinduction

## Abstract

In this article, we proposed a facile protocol to functionalize porous calcium phosphate ceramics (PCPC) using dietary tea polyphenols (TP). TP molecules was attracted and anchored by Ca^2+^ ions from the surface of CPC. These TP molecules modulated the nucleation and crystallization of calcium phosphate nanorods assemblies on the surface of PCPC. Our results prove that these calcium phosphate nanorods assemblies accompanies functional groups of TP make PCPC/TP effectively promote proliferation and differentiation of bone mesenchymal stem cells (BMSCs). We inferred that these calcium phosphate nanorods assemblies might change the surface microenvironment of PCPC, which is critical to promote the proliferation and differentiation of BMSCs. Compared with naked PCPC, PCPC/TP obviously increased BMP2, ErK/MAPK and JNK/MAPK level and mineralization capacity of cells (ALP level).

## Introduction

Clinical bone defects are common orthopedic diseases. Bone defects are usually treated by transplantation, including autologous bone and artificial bone material. Autologous bone graft often brings new trauma to the patient, and the amount of bone graft is limited. So, it cannot meet the needs of greater trauma. Artificial bone material as a popular material for bone tissue engineering can repair a large number of bone defects. Therefore, artificial bone material is the most ideal bone graft material. But it is still in the experimental stage, not widely used in clinical. Calcium phosphate ceramics (CPC) are frequently used as bone substitute materials because of their chemical similarity to bone mineral and their desirable characteristics, such as high bioactivity, osteoconduction and even osteoinduction [1–6]. It is reported the porous calcium phosphate can strongly adsorb bone-growth related proteins [7, 8], such as bone morphogenetic protein-2 (BMP2). The osteoinducctive capacity of CPC may due to the activation of intracellular BMP/Smad pathway [9, 10], which can be strongly affected by surface topography, architecture, degradation properties and ionic microenvironment [8, 11–17]. The surface properties of CPC may also affect the expression of osteocalcin (OCN), osteopontin (OPN) and alkaline phosphatase (ALP) for bone mesenchymal stem cells (BMSCs), which are essential to bone formation and reparation [18–20]. Therefore, it is very important to develop surface functional CPC, especially used for promoting proliferation and differentiation of BMSCs. BMSCs are derived from non-hematopoietic tissues and have strong proliferative capacity and multi-directional differentiation potential. The capacity of osteogenic differentiation is a key issue in the study of bone defect repair [21].

Green tea polyphenols (TP) can increase bone mineral density by inducing apoptosis of osteoclast to prevent bone resorption [22, 23]. Our former work shown that TP molecules have strong ability to modulate the crystallization of minerals [24]. Herein, in this article, we proposed a facile protocol to functionalize porous calcium phosphate ceramics (PCPC) using TP. We inferred that such calcium phosphate nanostructures might change the surface microenvironment of PCPC, which could promote the proliferation and differentiation of BMSCs.

## Materials and methods

### PCPC and PCPC/TP

PCPC were provided by National Engineering Research Center for Biomaterials, Sichuan University, Chengdu, China. PBCP were assemble through H2O2 foaming and sintered at 1100 °C following the similar processing method mentioned before [25].

**Scheme 1.**
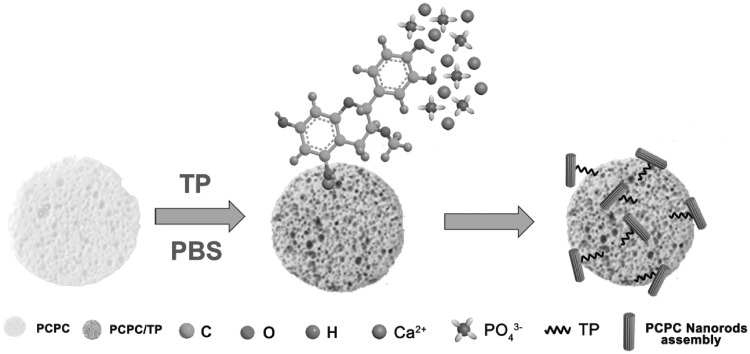
Schematic process of TP functionalized PCPC.

Green TP functionalized porous calcium phosphate (PCPC/TP) was prepared as shown in Scheme 1. Firstly, PCPC disk was put into 20 ml phosphate buffer solution (PBS, 0.01 M). Then, 5 mg TP was dissolved into the PBS solution. The system was set at 4 °C for 24 h. After that, the PCPC disk and the solution were transferred into a 50 ml autoclave and reacted at 200 °C for 4 h. Finally, TP modified PCPC disk (color changed from white into brown) was collected and rinsed. Prior to further application, TP modified PCPC disk was sterilized by γ-ray radiation.

### Isolation and culture of BMSCs

BMSCs were isolated from bone marrow new born (5 days) Sprague Dawley (SD) rats [26]. The rats were soaked in 75% ethanol for 3–5 min, cut off the legs and placed it in PBS solution for use. Then cut off the femur epiphysis at both ends of the tibia, flushed cells from the marrow cavity using F12/DMEM medium. Finally, we dispersed the cells and inoculated the cells into the flask. After 48 h, we exchanged the medium of primary cells, and we used the fourth generation cells for this experiment.

### Cell adhesion to PCPC and PCPC/TP

BMSCs were cultured with PCPC and PCPC/TP (washed with PBS thoroughly) in an incubator (5% CO_2_ atmosphere) at 37 °C for 6 h. After that, they were rinsed by PBS to eliminate uncombined cells. Then, the cells were stained with TRITC conjugated-phalloidin (1:200, Sigma) and anti-BMP2 antibody (1:1000 Abcam ab6285), and observed under a confocal laser scanning microscopy (CLSM, FV10i, Olympus, Japan). Moreover, cell adhesion was further observed by scanning electron microscope (SEM, S4800, Hitachi, Japan).

### Proliferation of BMSCs in the leach liquor of PCPC, PCPC/TP and normal medium

MTT ([3-(4, 5-dimethylthiazol-2-yl)-2, 5-diphenyl tetrazolium bromide]) assay was used to evaluate the proliferation of BMSCs in the leach liquor of PCPC, PCPC/TP and normal medium at different time. Firstly, BMSCs were seeded in a density of 5000 cells per well (96 well plate) and cultured for 24 h. Subsequently, the culture medium was removed and replaced by the leach liquor of PCPC, PCPC/TP and normal medium. MTT time point was set at 6, 12 and 24 h. At each time, 20 μL of MTT solution (5 mg/ml in PBS) was added into each well. After 4 h treated, the MTT medium was removed. Finally, 150 μl of DMSO was added into each well. Quantitative detection was performed on a microplate reader at the wavelength of 490 nm.

### Alizarin red staining and ALP activity detection

BMSCs were cultured with the leach liquor of PCPC and PCPC/TP and normal medium for 14 days. Then, they were washed with PBS for three times. After that, they were fixed by using paraformaldehyde for 10 min. After washing three times by PBS, they were stained by using 0.1% alizarin red for 30 min in a 37 °C incubator. Finally, they were washed with PBS for 3 times. And ALP activities were measured using the Lab Assay ALP kit (Wako). We calculated the percentage of alizarin red stained calcified nodules areas using the image J software.

### Western blot

After 3 days cultured with materials, the cells were collected, and lysed with RIPA buffer (Beyotime, China). Then, the protein concentration was detected by BCA protein assay kit (Pierce, IL, USA). Equal aliquots of protein (15 ul) were heated at 100 °C for 10 min, and been fractionated using 10% SDS-PAGE gels. Then, they were put on PVDF films, and treated by TBS-T (with 1% BSA) for 2 h. After that, they were rinsed thoroughly, incubated overnight with primary antibodies to OCN (1:200 Santa Cruz, sc-390877), OPN (1:200 Santa Cruz, sc-21742), p-ErK (1:200 Santa Cruz, sc-81492) and JNK1 (1:200 Santa Cruz, sc-4061) at 4 °C, respectively. Subsequently, they were cultured with secondary antibody (diluted 1:5000 Earthox) for 2 h. The kinase activities of OPN/Actin, JNK1 MAPK/Actin and p-ErK MAPK/Actin were evaluated according to the reported method [27].

### Immunocytochemistry

The leach liquor of calcium phosphate was added to well plate (0.1 mg/ml). Then, BMSCs were trypsinized, suspended and incubated in leach liquor of calcium phosphate for 24 h at 37 °C in incubator with 5% CO_2_ atmosphere. Cells were also incubated with primary anti-p-ErK/anti-JNK1 antibodies (1:50 Santa Cruz) overnight at 4 °C. Then, they were washed three times with PBS. After that, they were incubated for 1 h with Alexa Fluor 488 (1:1000 ThermoFisher Scientific) and washed three times with PBS. Subsequently, cells were incubated with TRITC conjugated-phalloidin (1:200, Sigma) for 1 h at 37 °C. After washing, cells were incubated with Hoechst 33258 for 5 min, then washed for three times before further characterization.

## Results

**Figure 1.**
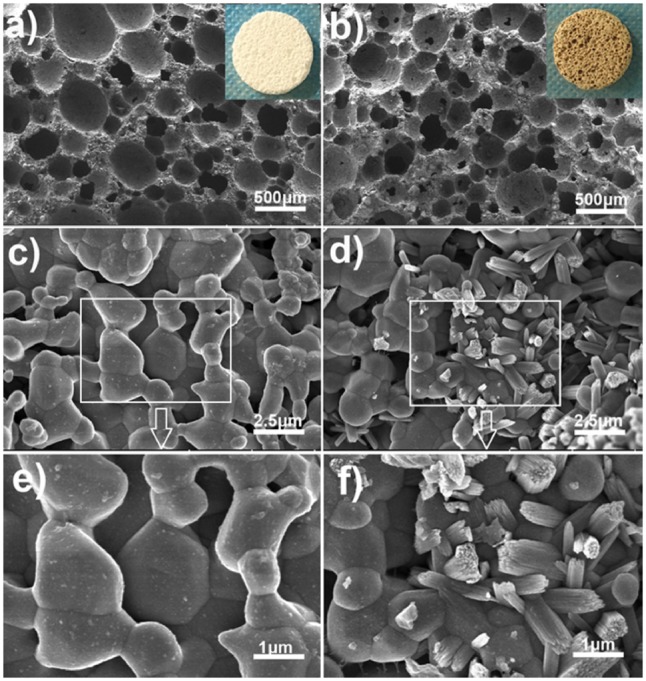
SEM images about the porous (**a**, **b**) and inside the porous (**c**–**f**) of PCPC (a, c, e) and PCPC/TP (b, d, f).

Figure 1 shows the porous structure and surface details of PCPC and PCPC/TP. Figure 1a (PCPC) and 1b (PCPC/TP) reveal that both PCPC and PCPC/TP having macropores (1–2 mm) and micropores (0.1–2 μm). This suggest TP functionalized PCPC/TP doesn’t change the original porous structure of PCPC. However, Fig. 1c–f show their surface morphology are much different. The rough surface of PCPC is only composed by crystal grain with size range of 1–5 μm (Fig. 1c and e). While the surface of PCPC/TP is distributed with nanorods assembly (Fig. 1d), the magnified image in Fig. 1f further present the detailed structure of those nanorods are about 1.5 μm long and 20 nm in diameter.

**Figure 2.**
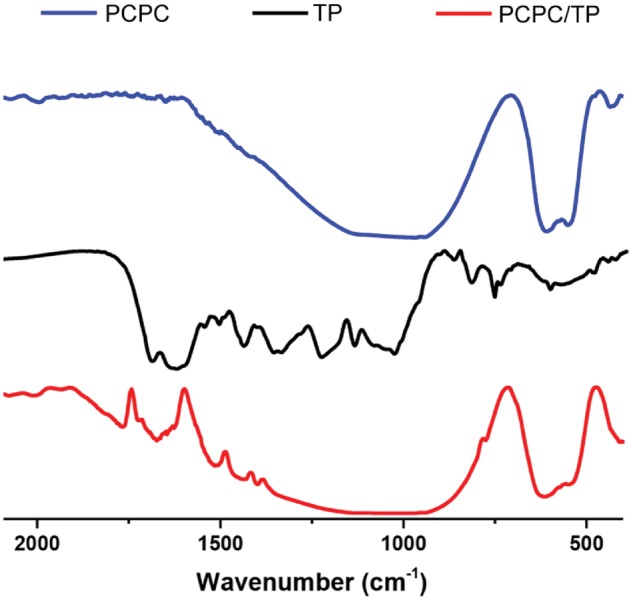
Spectra of PCPC, TP and PCPC/TP.

As we reported previously, TP molecules have strong ability to modulate the crystallization of minerals [24]. The above results from SEM images present in Fig. 1 indicate that TP molecules played an important role in modifying the morphology and structure of PCPC. This might because of the interactions between TP and PCPC, such as H-bonding between the phenol group of TP and phosphate groups of PCPC and electrostatic interactions between phenol group of TP and Ca^2+^. The FT-IR absorptions at 2878, 1670 and 1398 cm^−1^ (characteristic absorptions of TP, Fig. 2) reveal that the incorporation of TP into the obtained PCPC/TP. Thermogravimetric analysis (TGA, NETZSCH STA 449 C DSC/DTA-TG analyzer scanning from 30 to 600 °C under an air atmosphere, Supplementary Fig. S1) of the samples indicate that PCPC/TP contains 1.87 wt% of TP. Furthermore, the new endothermal peak appears at 475 °C in the differential scanning calorimetry thermogram (DSC, Supplementary Fig. S1) of PCPC/TP, indicative of the enhanced thermal stability of TP. This suggest that the incorporated TP molecules are not only been adsorbed, but might been solidified by PCPC/TP.

**Figure 3.**
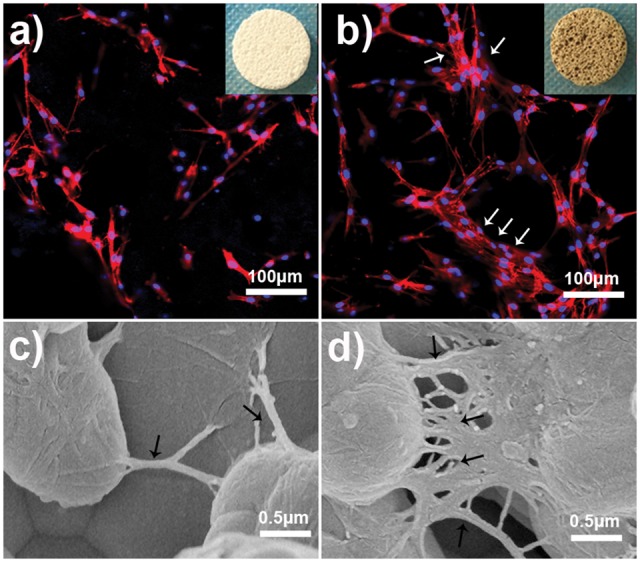
CLSM observation for BMSCs growth on PCPC (**a**) and PCPC/TP (**b**) at 1 day. SEM observation for BMSCs adhesion onto PCPC (**c**) and PCPC/TP (**d**).

Figure 3 presents the CLSM and SEM images of BMSCs cultured on PCPC (Fig. 3a and c) and PCPC/TP (Fig. 3b and d). From CLSM images (indicated by arrows in Fig. 3a and b), it can find that PCPC/TP could attract more BMSCs to adhesion and spreading on it than PCPC. SEM images in Fig. 3c and d further reveal that the microfilament structure of cells (indicated by arrows in Fig. 3c and d) seeded on PCPC is less developed than those of PCPC/TP. Moreover, this difference may also suggest that morphology striking of cells on PCPC and PCPC/TP is different. Morphology of cells on PCPC/TP is more stretch than those of PCPC. The significant differences on shape and cell density may because the surface nanostructure as shown in SEM images has been changed, which may be critical in adsorbing proteins [28]. As shown in Scheme 1, TP molecules will firstly be attracted by Ca^2+^ ions form the surface of CPC. Subsequently, these anchored TP molecules would modulate the nucleation and crystallization of CPC nanostructures on the surface of PCPC. We inferred that such calcium phosphate nanostructures might change the surface microenvironment of PCPC, which could promote the proliferation and differentiation of BMSCs.

**Figure 4.**
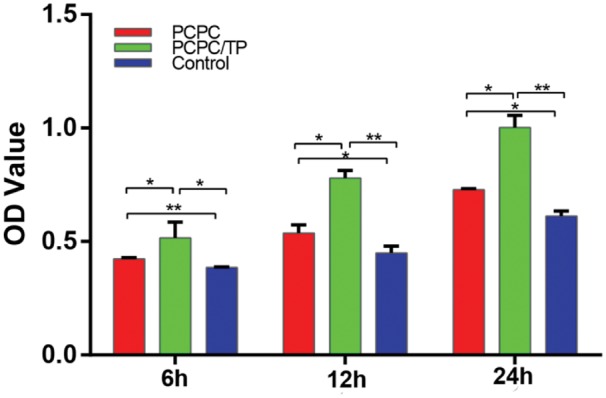
BMSCs cell viability studies and statistical analysis result of osteoblasts cultured under PCPC and PCPC/TP at 6, 12 and 24 h (*P < 0.05, *n* = 3).

From the morphology change result showed above, it can be inferred that PCPC/TP may promote cell proliferation. In order to investigate the effect of PCPC/TP on cell proliferation of BMSCs, we used MTT method to study in different times. Figure 4 presents the detailed proliferation of BMSCs in PCPC, PCPC/TP and control groups. It is obviously that proliferation of BMSCs cells were significantly promoted by PCPC/TP, compared with the other two groups. Moreover, this promote trend became more obvious with time increased.

**Figure 5.**
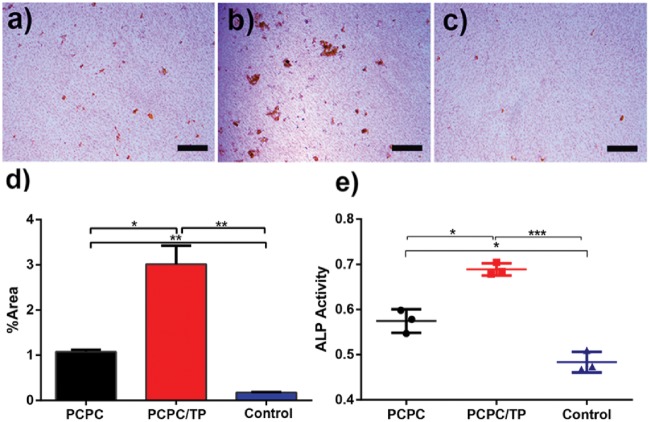
Alizarin red staining images of BMSCs cultured in leach liquor of PCPC (**a**), leach liquor of PCPC/TP (**b**) and normal medium (**c**) for 14 days. The graph of the percentage of calcified area and statistical analysis result is **d**. ALP activity in BMSCs was treated with leach liquor of PCPC, leach liquor of PCPC/TP and normal medium after 14 days. The scale bars are 400 μm. (*P < 0.05, *n* = 3).

The normal groups (Fig. 5c), leach liquor of PCPC (Fig. 5a) and PCPC/TP (Fig. 5b) cultured in BMSCs differentiation medium for 14 days, followed by alizarin red S staining assay. The results showed that the staining level of calcified plaque treated with PCPC/TP is significantly higher than others through the calcified area analysis (Fig. 5d). The ALP activity were examined via ALP kit, the results (Fig. 5e) showed that the activity of ALP cultured in leach liquor of PCPC and PCPC/TP was greatly higher than normal group. In addition, the leach liquor of PCPC/TP was higher than PCPC.

**Figure 6.**
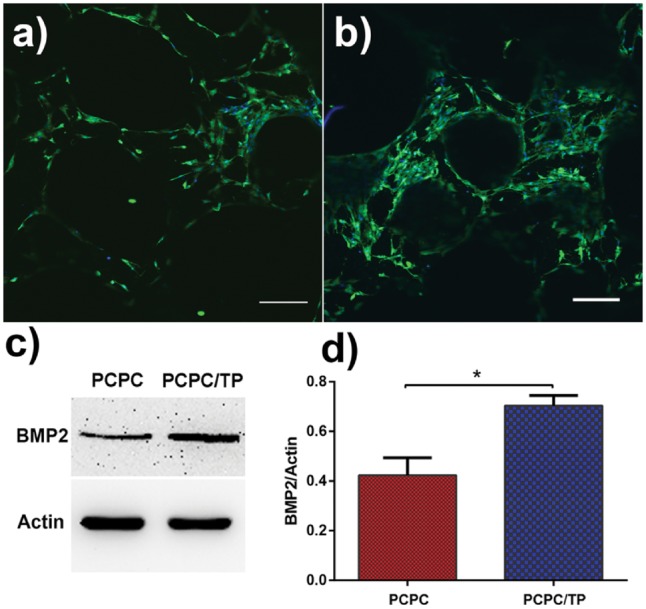
CLSM observation the level of BMP2 for BMSCs growth on (**a**) PCPC and (**b**) PCPC/TP. WB analysis for activation of (**c**, **d**) for BMSCs cultured in PCPC and PCPC/TP. The scale bars are 200 μm.

The activities of BMP2 were examined via immunofluorescence (Fig. 6a and b), western blot (WB) (Fig. 6c) and WB analysis (Fig. 6d). It was found that PCPC/TP increased the level of BMP2 compared with naked PCPC.

**Figure 7.**
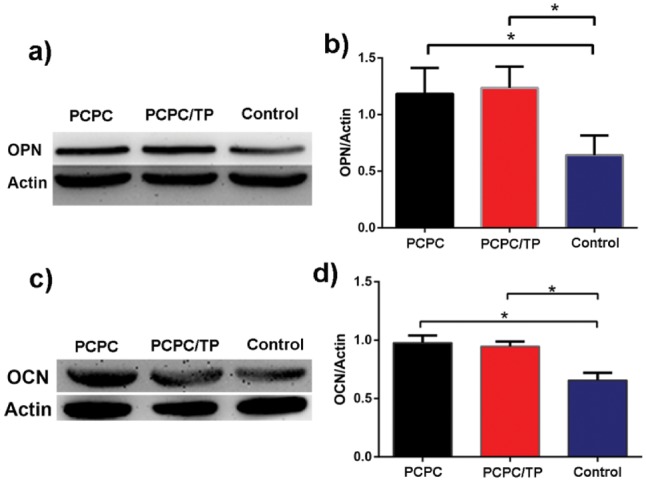
WB assay and quantification analysis result of the level of OPN (**a**, **b**) and OCN (b, **d**). Actin used as control (*P < 0.05, *n* = 3).

The level of OCN (Fig. 7a and b) and OPN (Fig. 7c and d) was observed by WB analysis. Both PCPC and PCPC/TP can increase their level of protein compared with normal group. But the difference between PCPC and PCPC/TP was not statistically significant.

**Figure 8.**
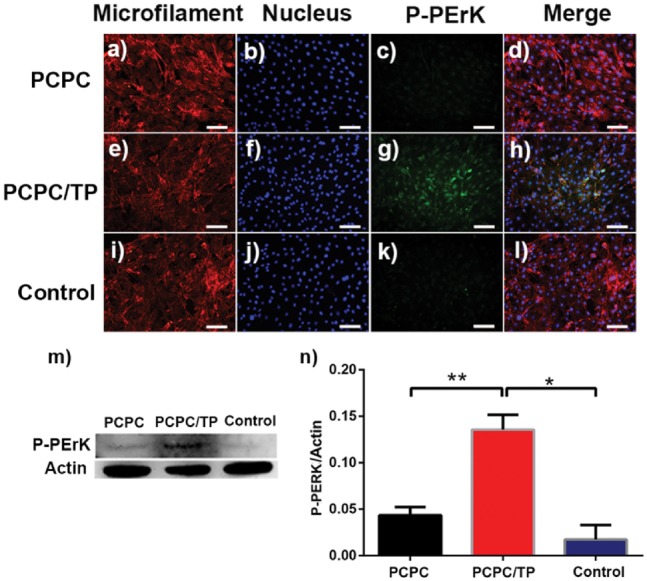
Fluorescence images of the level of P-ErK. BMSCs in images (**a**–**d**) were cultured in leach liquor of PCPC medium, (**e**–**h**) was cultured in leach liquor of PCPC/TP and (**i**–**l**) was cultured in normal medium. Cytoblast (c, g, k) were stained with hoechst 33258 (blue). The scale bars are all 100 μm. WB assay (**m**) and quantification analysis (**n**) result of the expression of P-ErK. Actin used as control. (*P < 0.05, *n* = 3).

**Figure 9.**
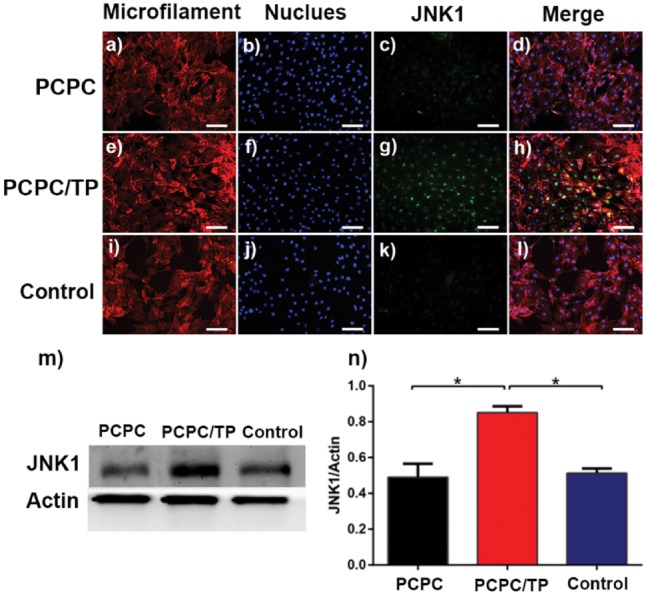
Fluorescence images of the level of JNK1. Osteoblast cells in images (**a**–**d**) were cultured in leach liquor of PCPC medium, (**e**–**h**) was cultured in leach liquor of PCPC/TP and (**i**–**l**) was cultured in normal medium. Cytoblast (c, g, k) were stained with hoechst 33258 (blue). The scale bars are all 100 μm. WB assay (**m**) and quantification analysis (**n**) result of the level of JNK1. Actin used as control. (*P < 0.05, *n* = 3).

Activities of p-ERK1/2 and JNK1 have been determined by WB and fluorescence (Figs. 8a–l and 9a–l). The results indicate that PCPC/TP has increased level of p-ERK1/2 (Fig. 8m and n) and JNK1 (Fig. 9m and n). While naked PCPC did not obviously increase the level of ERK1/2 and JNK1 compared with the control group.

## Discussion

Previous literatures prove that calcium phosphate can induce bone formation, promote the osteogenic differentiation of MSCs, and modulate the crystallization of minerals [8, 29, 30]. Herein, we aimed to make PCPC exhibits more excellent osteoinduction using TP to modify its surface structure. Our protocol can use the merits of TP combined with the advantages of calcium phosphate. It is reported the porous calcium phosphate can strongly adsorb bone-growth related proteins [7, 8], such as BMP2. Our result showed that PCPC/TP further increased the level of BMP2 compared with PCPC (Fig. 5). Bone morphogenic proteins (BMPs), such as BMP2, are members of transforming growth factor (TGF) which can induce the formation of bone [31]. BMP2 can promote osteoblasts differentiation and inhibiting its apoptosis. It can also promote relative osteogenic factor expression, such as Cbfa1, COL1 and ALP, which play a key role in osteoblasts differentiation [32]. Our results from WB assay and immunofluorescence staining suggest that PCPC/TP is more beneficial to osteogenic differentiation than pure PCPC. The reason may due to the enhanced adsorptive protein capacity by incorporated TP and the new formed calcium phosphate nanorods.

It is known that the activating MAPK signaling pathways can induce osteogenic differentiation of BMSCs [33–35]. Our results from WB assay and immunofluorescence staining suggest that PCPC/TP can efficiently increase the level of ErK/MAPK and JNK/MAPK, two major intracellular MAPK signals. This may due to TP molecules and new formed calcium phosphate nanorods provided more functional groups (TP) and higher surface area (calcium phosphate nanorods) to attract the beneficial molecules. Whether these new functional groups have a promoting effect on the BMCSs has not been studied yet. Therefore, in the next research we can study in detail the function of these functional groups on the cells, in order to prove whether these functional groups cause cell proliferation and differentiation.

OPN and OCN are specific products of osteoblast activity, in which OPN is a sign of osteoblast differentiation and maturation, and OCN mainly appears in the mineralization stage, which is a sign of calcification and maturation of osteoblast extracellular matrix [36–40]. Our results from WB assay suggest PCPC/TP is beneficial to osteogenic differentiation compared with control group, but there was no statistically significant difference between PCPC and PCPC/TP in OCN and OPN level. These results may suggest that PCPC/TP did not increase the level of OCN and OPN, but highly increased ALP activity (Fig. 5) and BMP2 level (Fig. 6).

## Conclusion

In this article, we proposed a facile protocol to functionalize PCPC using TP. TP molecules was attracted and anchored by Ca^2+^ ions from the surface of CPC. These TP molecules modulated the nucleation and crystallization of PCPC nanorods assemblies on the surface of PCPC. Our results prove that these PCPC nanorods assemblies accompanies functional groups of TP make PCPC/TP effectively promote proliferation and differentiation of BMSCs. We inferred that these PCPC nanorods assemblies might change the surface microenvironment of PCPC, which could promote the proliferation and differentiation of BMSCs. Compared with naked PCPC ceramics, PCPC/TP obviously increased BMP2, ErK/MAPK and JNK/MAPK level and mineralization capacity of cells (ALP level).

## Supplementary data

Supplementary data are available at *REGBIO* online.

## Supplementary Material

Supplementary Data
