# Supplementary figures and images for: Promoting proliferation and differentiation of BMSCs by green tea polyphenols functionalized porous calcium phosphate
Source: Regen Biomater. 2017 Dec 11;5(1):35–41. doi: 10.1093/rb/rbx031 (PMC5798040; doi:10.1093/rb/rbx031)

## Supporting Information

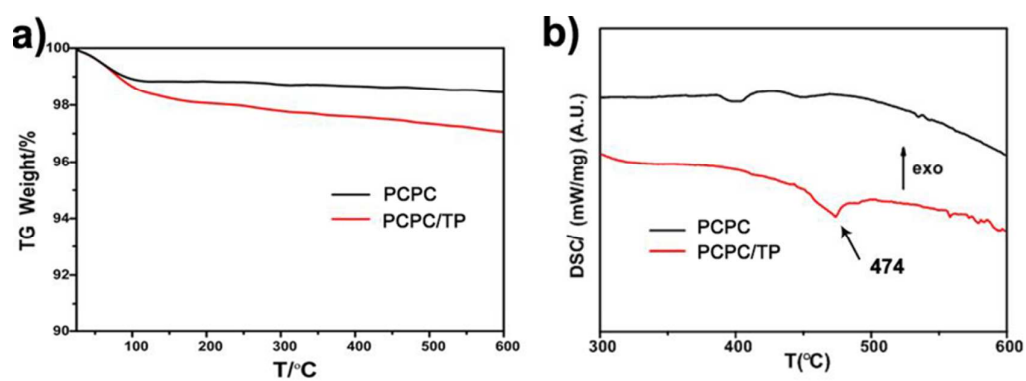

Fig.S1 TG (a) and DSC (b) analysis of PCPC and PCPC/TP samples.

Supplement: Supplementary Data [file rbx031_supp_figure.pdf]
